# Increasing Couriers’ Job Satisfaction through Social-Sustainability Practices: Perceived Fairness and Psychological-Safety Perspectives

**DOI:** 10.3390/bs13020125

**Published:** 2023-02-02

**Authors:** Qiwei Pang, Mingjie Fang, Lu Wang, Kena Mi, Miao Su

**Affiliations:** 1 College of International Economics & Trade, Ningbo University of Finance & Economics, Ningbo 315175, China; 2 Department of Economics, Sejong University, Seoul 05006, Republic of Korea; 3 Department of Logistics, Service & Operations Management, Korea University Business School, Seoul 02841, Republic of Korea; 4 School of Business, Zhejiang University City College, Hangzhou 310015, China; 5 The Graduate School of Technology Management, Kyunghee University, Yongin 17104, Republic of Korea

**Keywords:** COVID-19 pandemic, equity theory, job satisfaction, Maslow’s hierarchy-of-needs theory, psychological-safety theory, social-sustainability practices

## Abstract

Due to the spike in online-retail demand during the pandemic, couriers confront increased workload and safety concerns, posing significant social-sustainability challenges for courier companies. This study explores the impact of social-sustainability practices on couriers’ job satisfaction in the context of the COVID-19 pandemic. We designed the research model from the theoretical lens of Maslow’s hierarchy of needs, equity, and psychological-safety theories. We collected the views of 428 couriers from the Chinese market, where there is a developed e-commerce industry. The structural-equation-model analysis results found that social-sustainability practices such as working environment, working conditions, health and safety, education, and training positively affected the job satisfaction of couriers during the pandemic through the mediators (psychological safety and perceived fairness). These findings provide empirical recommendations for improving employees’ job satisfaction in courier companies during COVID-19 and addressing the social-sustainability issues of courier companies.

## 1. Introduction

COVID-19 impacted more than 200 countries and regions globally, causing an unprecedented pandemic [1,2] with nearly 592 million confirmed cases and 6.44 million deaths worldwide as of August 19, 2022 [3]. Despite the current development and use of the COVID-19 vaccine, COVID-19 continues to be the dominant health threats worldwide [4]. As an important measure to combat COVID-19, social distancing gradually became recognized as a new rule to guide daily life [5,6,7]. In addition, one of the most important and effective measures included encouraging consumers to use e-commerce for shopping, which led to increasing demand for e-commerce [8,9,10,11]. Since the necessary support for the e-commerce industry comes from courier companies, including receiving, sorting, packaging, transportation, distribution, and other processes [12], the results of COVID-19 and social distancing have led to a shortage of couriers. This shortage is in the courier companies, including an increase in the workload of the original couriers [13] and creating unprecedented challenges for courier companies and the industry [14,15,16].

According to the New York Times, Forbes, and other media reports, during the COVID-19 pandemic, the pressure on courier companies far exceeded the workload that couriers could bear [13,17]. During the pandemic, the safety and health of Chinese couriers have been severely compromised. According to a Workers’ Health and Safety Center survey, couriers have worked an average of 12 h a day, 6 days a week, since the outbreak of COVID-19, and the number of work-related injuries among couriers has increased by 43% [18]. Furthermore, during the COVID-19 pandemic, couriers who exceeded their workload reported extremely depressed job satisfaction, and productivity declined [14]. In addition, in receiving, sorting, packaging, transportation, and distribution, COVID-19 causes a hidden danger to the health and safety of couriers working together [19]. This unsafe working environment threatens the employees’ sense of job security, decreasing the employees’ overall satisfaction [20]. Hence, due to the increase in demand for e-commerce caused by social distancing [9] and the direct impact of COVID-19 on courier companies’ employees [19], courier companies have serious social-sustainability issues.

The concept of sustainable development has gradually changed the strategy and management methods of enterprises by considering the three dimensions of the environment, economy, and society. However, scholars have focused primarily on the environmental and economic dimensions [21,22], and social sustainability has only been widely discussed in the last two decades and has been incorporated into sustainability analysis [23,24]. Social sustainable development refers to the provision of appropriate working conditions by protecting workers from exploitation, maintaining a healthy and safe environment with fair wages and equal treatment, providing employee training, and encouraging freedom of association [25]. According to Valiance, et al. [26], society needs to achieve sustainable development through the continuous creation of social capital, the promotion of social equity, and the satisfaction of basic human needs. Meanwhile, Govindan, et al. [27] note in their study that social-sustainability management for businesses is a challenging task and that ensuring a safe working environment, fair working conditions, training, and health care for employees through socially sustainable practices is necessary.

Given its important and abundant relevance, research on social sustainability has grown dramatically, including research into the social sustainability of companies based on a supply chain management perspective [24,28,29,30,31,32,33,34,35,36]. The main emphasis is on ethical production, the need for positive interaction between all parties in the supply chain, and the importance of companies establishing a sustainable social strategy. Meanwhile, other scholars have studied the impact of corporate social sustainability on corporate performance [37,38]. For example, Mani, Jabbour and Mani [38] verified that social sustainability positively impacts a company’s operational performance. Furthermore, they argue that emerging economies achieve growth in performance gains by improving social sustainability.

Studies also explore firms’ social sustainability based on corporate social responsibility [23,39,40]. For example, Toussaint, Cabanelas and Blanco-Gonzalez [23] advocated in their research on the food value chain that companies should improve their transparency and market awareness. In addition, they said companies should fulfill corporate social responsibility by developing good social standards to win the trust of consumers and distributors and achieve social sustainability for the company. Overall, research on corporate social sustainability mainly focuses on supply chain perspectives, company performance, corporate social responsibility, etc. [22,35,41]. However, research on socially sustainable practices at the employee level of the enterprise is insufficient; most research has focused on the level of moral production. In particular, there is very little research on job satisfaction about social sustainability. Furthermore, under the sudden attack of COVID-19, consumers’ consumption habits have undergone major changes [9,11], and the social-sustainability problems of courier companies that support e-commerce transactions [12] have yet to be solved. Hence, courier companies urgently need to improve employee job satisfaction through social-sustainability practices adjustments in response to the COVID-19 pandemic or other contingencies that may lead to social-sustainability issues.

China has a vast population and a significant number of employees, but their social rights and interests have not been addressed seriously. In addition, underdeveloped nations pay minimal attention to socially sustainable employment practices [42]. China’s corporate social responsibility is still in its infancy, according to the study, and more empirical research is urgently required in this field [43]. Moreover, during emergencies such as the epidemic, Chinese enterprises pay little regard to the rights and interests of their employees, which makes empirical study during the pandemic all the more vital.

Therefore, to solve the social-sustainability problem of courier companies, this study adopts a theory-driven approach to social-sustainability practices to solve the social-sustainability issues of courier companies, thereby filling the knowledge gap in this area. Specifically, based on Maslow’s hierarchy of needs [44], equity [45], and psychological-safety theories [46], this study establishes a targeted model to explore the deep causes affecting job satisfaction in courier companies during the COVID-19 pandemic. An important way to increase job satisfaction is to meet the needs of employees [47], especially when disasters compromise the vital needs of employees [48]. Thus, this study uses Maslow’s hierarchy of needs to analyze the factors that drive certain needs of employees. Meanwhile, organizations have widely used equity theory to study employee responses to reward distributions and job satisfaction [49,50,51]. In addition, this study combines psychological-safety theory, an important factor in influencing employee psychology and behavior in the company, especially when disasters such as COVID-19 are happening [52,53,54].

Multiple contributions are made by this study. This study first investigates the causal relationship between company social-sustainability initiatives and employee satisfaction, thereby contributing to the literature on employee psychology and behavior. This work contributes to the advancement of both theoretical and empirical research in the field of behavioral science. This study specifically examines the formation process of employee satisfaction via the lens of Maslow’s hierarchy-of-needs theory, equity theory, and psychological safety theory. Lastly, from the perspective of management application, this study gives several employee satisfaction management improvement measures for ex-press logistics companies.

The structure of the remaining four sections of this study is as follows. Section 2 elaborates on the theoretical background, proposed research models, and hypotheses. Section 3 details the survey methodology, data collection, and data characteristics. We present the analysis results in Section 4 and summarize the research contributions and future research directions in Section 5.

## 2. Literature Review and Hypothesis Development

### 2.1. Theoretical Background

**Figure 1:**
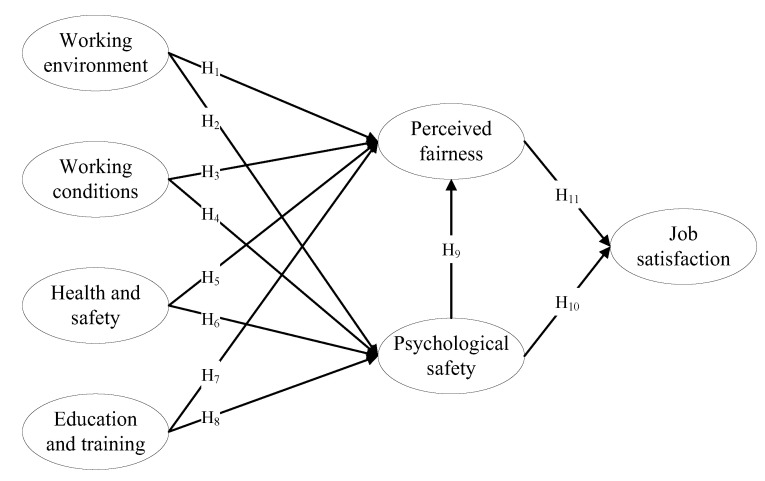
Theoretical Framework.

This study adopted Maslow’s hierarchy of needs, equity, and psychological-safety theories to construct a research model (Figure 1) to analyze the deep causes of job satisfaction in courier companies under the impact of the COVID-19 pandemic. Table 1 describes each theory’s paradigm, basic assumptions, and applications to the model.

Maslow divides human needs like a ladder from low to high into five levels: physiological, safety, social, esteem, and self-actualization [44]. Researchers and organizations continue to use this theory to understand human behavior and apply it widely to business and corporate management [47,55,56]. Therefore, this study further divided the Maslow hierarchy into three subcomponents based on the needs of employees: (1) physiological, safety, and social needs consisting of education and training, health and safety, working environment, working conditions, and psychological safety; (2) esteem needs that include perceived fairness; (3) self-actualization that consists of satisfaction. In addition, disasters such as COVID-19 compromise and amplify human needs [48], thus necessitating studying social-sustainability issues facing companies at the level of employee needs [55].

Equity theory [45] is also widely used in the field of corporate management [51] as a classic theory that can explain employee job satisfaction [50]. Equity theory holds that any employment relationship is an exchange between an employee and an organization [57]. In this relationship, the ratio (i.e., perceived fairness) between the outcomes received by the employees and their inputs determines the satisfaction of the employees [50]. For example, Klein [57] asserts that if the outcomes obtained by employees (e.g., working conditions which include income and rewards, working environment, health and safety, education, and training) do not match the inputs, it will lead to a decrease in employee satisfaction. In this study, changes in consumption patterns during the COVID-19 pandemic increased couriers’ stress [14], undermining their basic needs and posing a serious risk to their health [20]. The emergence of such social-sustainability issues at the employee level increases employee inputs, so we needed to introduce an equity theory to study the antecedents affecting job satisfaction.

Furthermore, the COVID-19 pandemic increased couriers’ stress and anxiety, thereby reducing their psychological safety [14]. For example, Ahmad, Donia and Shahzad [52] believe that psychological safety for employees means they can work in a safe environment and express their feelings. Meanwhile, many studies on company management have shown that employee psychological safety is an important factor influencing employee behavior and satisfaction [53,54]. Moreover, the key to improving employees’ psychological security is reducing harsh work requirements, meeting employees’ needs, and making employees think that the company is ethical [52,54]. Therefore, this research combined psychological-safety theory and explored the antecedents affecting couriers’ job satisfaction by studying the needs of employees, thereby carrying out sustainable social practices.

### 2.2. Hypothesis Development

#### 2.2.1. The Impact of Couriers’ Working Conditions and Working Environment on Perceived Fairness and Psychological Safety

The working environment and working conditions, as basic employee needs, are important indicators of a company’s social sustainability [39,58]. The working environment includes the employees’ physical (e.g., lighting, ventilation, and leisure facilities) and psychological working environments (e.g., public discussion and psychological comfort) [59,60]. Working conditions include the company’s benefits to employees, such as remuneration, working hours, contract systems, and other employee benefits [58]. Through optimization measures such as increasing salaries, shortening working hours, and creating a good and safe working environment, employees can obtain a fair perception when they compare the treatment of other companies or other occupations in society at work [61,62]. In this research, the direct and indirect effects of the pandemic increase couriers’ self-inputs. Thus, according to equity theory, employees perceive fairness if their outcomes increase to balance their inputs and outcomes [50]. Therefore, improving employees’ working environment and working conditions will make employees feel that the outcomes increased [63] and, thus, induce fairness.

In addition, Ahmad, Donia and Shahzad [52] argue that if organizations try to meet the needs and care of their employees so that they think the organization is ethical, it will lead to a greater psychological sense of security. Working conditions and working environments are among the most basic and important physiological needs [44]. Undermining such basic needs compromises couriers’ psychological safety. However, during the COVID-19 pandemic, the working hours of couriers increased significantly, and the working environment during the sorting and packing process was also full of dangers. Changes in working conditions can increase employees’ worries about the future. Meanwhile, working in a dangerous work environment can also increase employees’ sense of insecurity, and even they will be afraid of spreading the disease to their families [16]. This feeling of unease can lead to employees’ distrust of the company and seriously damage the psychological safety of employees [52]. Therefore, we propose the following hypotheses:

**Hypothesis** **1** **(H1).** *The working environment positively impacted couriers’ perceived fairness during the COVID-19 pandemic.*

**Hypothesis** **2** **(H2).** *The working environment positively impacted couriers’ psychological safety during the COVID-19 pandemic.*

**Hypothesis** **3** **(H3).** *The working conditions positively impacted couriers’ perceived fairness during the COVID-19 pandemic.*

**Hypothesis** **4** **(H4).** *The working conditions positively impacted couriers’ psychological safety during the COVID-19 pandemic.*

#### 2.2.2. Direct Impact of COVID-19: The Impact of Couriers’ Health and Safety on Perceived Fairness and Psychological Safety

Hu, Yan, Casey and Wu [19] assert that employees in labor-intensive businesses are more vulnerable to the COVID-19 pandemic. In addition, since courier companies are a kind of labor-intensive enterprise [64], organizations cannot ignore the impact of COVID-19 on the health and safety of couriers. Couriers may be exposed to more health threats during their work than other occupations that have less contact with people. Therefore, this health threat can cause couriers to feel unfair, which stems from comparisons with other people or occupations in society. Moreover, Al-zawahreh and Al-Madi [63] believe that the health risks faced by employees are an important part of employees’ input in the employment relationship. In this study, the lower the health and safety of couriers, the higher the risk and increase in couriers’ inputs, reducing couriers’ perception of fairness. However, if the company guarantees couriers’ health and safety, it will reduce couriers’ input and make them feel fair. Furthermore, safety is one of humanity’s most basic and important needs [44].

Psychological activities such as employees’ insecurities, worries, and fears of working during the COVID-19 pandemic also cause tremendous psychological stress [65,66]. They worry about the damage from COVID-19 and the health of their families [16]. If the company does not take measures, it will also increase employees’ distrust of the company, making them work in a state of anxiety [52]. Therefore, ensuring the health and safety of couriers in the workplace (including implementing COVID-19 prevention measures) will reduce the psychological pressure of couriers and increase their sense of security. For example, courier companies in China set up rapid temperature testers and hand sanitizers at the entrance of the company to ensure the health and safety of the company’s couriers as much as possible. For these reasons, we put forward the following hypotheses:

**Hypothesis** **5** **(H5).** *Couriers’ health and safety positively impacted perceived fairness during the COVID-19 pandemic.*

**Hypothesis** **6** **(H6).** *Couriers’ health and safety positively impacted psychological safety during the COVID-19 pandemic.*

#### 2.2.3. The Impact of Education and Training on Couriers’ Perceived Fairness and Psychological Safety

The education and training of employees are also important indicators of social-sustainability practices [39,67]. For example, Trmcico, Demmings, Kniel, Wiedmann and Alcaine [1] assert that training employees on proper precautions during the COVID-19 pandemic can help reduce cross-infection among employees. In this study, the education and training of couriers include the conventional sense (e.g., job skills, related knowledge, etc.) and the non-conventional sense (e.g., providing epidemic prevention guidance for employees). Helping employees improve their job skills and enrich their work-related knowledge can increase employees’ expectations and rewards for the future. As a result, employees can feel a sense of fairness compared to other people and other occupations. Equity theory focuses on the inputs and outcomes of employees, which consider the ratio of their work inputs to outcomes [63]. Thus, according to equity theory, increasing couriers’ outcomes (i.e., regular training) and reducing couriers’ inputs (i.e., reducing the risks they face through epidemic-prevention guidance) will increase couriers’ perceived fairness [50].

Meanwhile, because courier companies are labor-intensive industries [64], the entry threshold is low. Therefore, employee education and training can meet employees’ increasing needs and trust in their companies’ ethics. Ma, Faraz, Ahmed, Iqbal, Saeed, Mughal and Raza [54] assert that psychological safety can improve employees’ motivation and actual performance, and that measures such as meeting employees’ needs, making employees feel cared for, and making the company trusted by employees can keep them in a state of psychological safety. Moreover, since the COVID-19 pandemic negatively impacts the psychology of employees in labor-intensive enterprises [65], certain epidemic prevention guidance could reduce employees’ fear of COVID-19 and promote employees’ mental health. Thus, we propose two more hypotheses:

**Hypothesis** **7** **(H7).** *Education and training have positively impacted employees’ perceived fairness during the COVID-19 pandemic.*

**Hypothesis** **8** **(H8).** *Education and training have positively impacted employees’ psychological safety during the COVID-19 pandemic.*

#### 2.2.4. The Impact of Couriers’ Psychological Safety on Perceived Fairness and Job Satisfaction

If employees are in a psychologically safe work environment, they feel safe taking risks [46]. For instance, Newman, et al. [68] found that employees with a low level of psychological security will think that if they proactively propose new ideas and learning innovative ways in the company, they may challenge the company’s established way of doing things, harm the interests of other employees, and put themselves at risk. In this study, the COVID-19 pandemic also brought great psychological pressure, causing couriers to perceive they were facing increased risks and inputs [20]. Thus, according to the equity theory [45], a low level of psychological safety will burden couriers with more inputs, making the inputs feel unfair. In contrast, a high level of psychological safety will make couriers receive greater benefits, reduce risk and inputs, and make them feel treated fairly.

In addition, the study related to green human resources by Moin, et al. [69] found a crucial impact of psychological safety on employee satisfaction. Psychological safety is a state of mind in which employees feel protected and share it openly without jeopardizing their career, job, or status, filling their organization with a positive work atmosphere [69]. Therefore, if employees are at a low level of psychological safety, they will be dissatisfied overall [70]. In contrast, organizations that foster a positive working atmosphere are more likely to produce satisfied and loyal employees [71]. Meanwhile, Widhoyoko and Sasmoko [56] argue that job safety is one of the most important factors influencing job satisfaction. Therefore, we propose the following assumptions:

**Hypothesis** **9** **(H9).** *Couriers’ psychological safety positively impacted perceived fairness during the COVID-19 pandemic.*

**Hypothesis** **10** **(H10).** *Couriers’ psychological safety positively impacted job satisfaction during the COVID-19 pandemic.*

#### 2.2.5. The Relationship between Perceived Fairness and Couriers’ Job Satisfaction

Job satisfaction is a widely studied concept in organizational behavior studies, often defined as the extent to which people enjoy their work [72]. It is one of the components of the highest stages (i.e., self-actualization) of Maslow’s hierarchy-of-needs theory [56]. Research shows that whether an organization is fair can affect employees’ job performance and job satisfaction [73,74]. In addition, Kim, et al. [75] found that positive changes in fair perception have a positive impact on job satisfaction and vice versa. This change is because employees who experience organizational justice tend to engage in positive work behaviors, such as continuous self-improvement, and are satisfied with their work [71]. In this study, couriers are satisfied with the organization and work if they are treated fairly, and they believe that the organization’s decisions are fair. In summary, if the couriers in this study have relatively strong perceived fairness, job satisfaction will increase, and vice versa, it will decline. Thus, this study proposes the following:

**Hypothesis** **11** **(H11).** *Perceived fairness positively impacted couriers’ job satisfaction during the COVID-19 pandemic.*

## 3. Methodology

### 3.1. Survey Design and Measurement Items

This study tested its hypotheses through an anonymous cross-sectional survey of couriers to study the social sustainability of courier companies during the COVID-19 pandemic. We designed the questionnaire in three parts. The first part introduced the background and purpose of the investigation to the courier company’s couriers. The second part asked couriers about 26 items to measure seven potential variables: work environment, working conditions, health and safety, education and training, perceived fairness, psychological safety, and job satisfaction (Table 2). In addition, to ensure the authenticity of the answers, we added two requests to the second part where interviewed employees chose “agree” or “disagree”. Finally, the third part asked respondents about their job and demographic information, including monthly income, work experience, gender, age, and education.

As Table 2 shows, we developed all items based on relevant expert inputs and existing studies to ensure the questionnaire’s effectiveness and adopted a seven-point Likert scale (1 = “strongly disagree,” 7 = “strongly agree”) to evaluate these items. We adopted four items relating to the working environment from Ali and Kaur [39]; Faulkner and Badurdeen [76]; and Trmcico, Demmings, Kniel, Wiedmann and Alcaine [1]. We also adopted four items to measure working conditions [58]. In addition, we chose three items relating to health and safety and four items for education and training from Ali and Kaur [39]; Rajak and Vinodh [67]; Trmcico, Demmings, Kniel, Wiedmann and Alcaine [1]; and Mani, Gunasekaran, Papadopoulos, Hazen and Dubey [24]. We also adopted four items of perceived fairness from Kim, Lin and Leung [75] and a three-item scale from Ahmad, Donia and Shahzad [52] and Lee, et al. [77] to detect psychological safety. Lastly, we measured employee job satisfaction using four items from Yuen, Loh, Zhou and Wong [72] and Sung and Hu [78].

Before we officially distributed the questionnaire, we conducted a pre-test on the courier company’s 20 couriers to ensure the accuracy of the survey results. The pre-tested couriers said they fully understood the items and purpose of the survey. However, two couriers interviewed said that the questionnaire did not clearly articulate some issues in the health and safety items. Thus, we added explanations and examples to the health and safety section.

### 3.2. Data Collection

We surveyed in China and paid for questionnaires completed with couriers of courier companies in Beijing, Shanghai, Guangzhou, Shenzhen, and Wuhan. The huge e-commerce market has led to the development of China’s logistics and courier companies [79], with a well-developed logistics infrastructure [80], and people have become accustomed to taking health and safety precautions during the COVID-19 pandemic [4]. Therefore, collecting the attitudes of couriers of Chinese courier companies is of great significance for studying the social sustainability of courier companies during the COVID-19 pandemic. We surveyed from 5 June 2021 to 10 September 2021 in the post-pandemic period of China. After excluding invalid surveys with incorrect screening items and too-short response times, we had 467 surveys for analysis (89.8% conversion rate).

This study examined the possibility of common method variance based on Harman’s single factor test proposed by Podsakoff, et al. [81]. The total variance in the single factor model is 30.87% (<40%), which means that in our data, the common method bias is not a problem. Moreover, we used the method proposed by Armstrong and Overton [82] and Chaudhuri and Holbrook [83] to check for non-responsive bias by return date. The results showed that there were no significant differences between the two groups.

### 3.3. Demographic Statistics and Work Experience

Table 3 shows the demographic data for the 467 respondents. The sample included 416 males (89.1%) and 51 females (10.9%)—the proportion of men was much greater than that of women. In addition, all of the respondents were under 46 years of age, with more than half of them under 25 (55.4%). Respondents’ age ranges were 25–35 (n = 140, 30.0%) and 36–45 (n = 68, 14.6%), with the majority of the interviewees between 25 and 55. Their education included high school or below (n =256, 54.8%), junior college (n = 178, 38.1%), and bachelor’s degree or above (n = 33, 7.1%). The respondents’ monthly income was less than CNY 3,000 (n = 43, 9.2%), 3,000–6,999 (n = 242, 51.8%), 7,000–10,000 (n = 147, 31.5%), and more than CNY 10,000 (n = 35, 7.5%). In terms of work experience, the couriers had from less than five to more than ten years: <5 years (n = 256, 54.8%), 5–10 years (n = 178, 38.1%), and >10 years (n = 33, 7.1%).

## 4. Results and Discussion

### 4.1. Measurement Model Assessment

We conducted a confirmatory factor analysis to test the model fit, reliability, and validity of the data (Table 4). We used a series of indicators, such as *χ^2^/df*, comparative fitting index (CFI), goodness-of-fit (GFI), Tucker–Lewis index (TLI), root mean square error (RMSEA), and standardized root mean square residual (SRMR), to evaluate the model fit, as suggested in previous studies [84,85]. Given that all its indices were within the cut-off range (*χ^2^*= 400.698, *df* = 278, *χ^2^/df* = 1.441, *p* < 0.05, CFI = 0.979, GFI = 0.940; TLI = 0.976, RMSEA = 0.031, SRMR = 0.033) [84,85], our measurement model has a good model fit. Additionally, all seven structures had a composite reliability above 0.7, supporting the model’s reliability [86]. We considered AVE and standardized factor loading as convergent validity measures. AVE values and standardized factor loads consistently exceeded 0.5, indicating good convergence validity [86]. In Table 5, the AVE values for various constructs are greater than the square of the correlation values with other structures, thus verifying the model’s discriminative validity [86].

### 4.2. Structural Model Assessment

**Figure 2:**
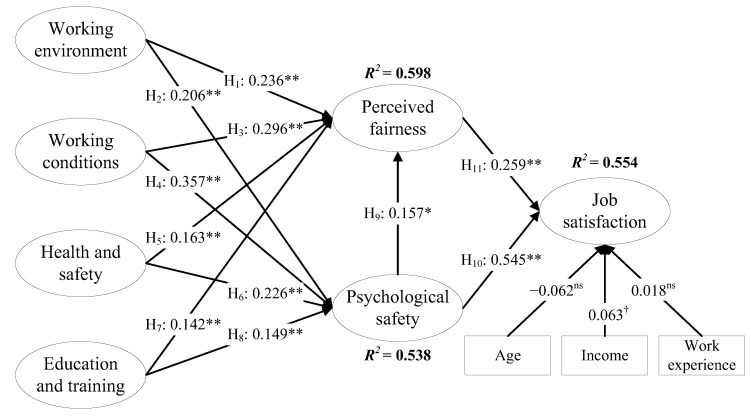
Results of Structural-Equation-Modeling Analysis. Note: ^†^ *p* < 0.1, * *p* < 0.05, ** *p* < 0.01, ^ns^ not significant; model fit indices: *χ^2^/df* = 1.592 (*p* < 0.05, *df* = 417); CFI = 0.961; TLI = 0.957; RMSEA = 0.036; SRMR = 0.046.

We used structural equation modeling to analyze the proposed hypotheses. Figure 2 illustrates the results (*χ^2^*= 663.864, *df* = 417, *χ^2^/df* = 1.592, *p* < 0.05, CFI = 0.961, GFI = 0.920, TLI = 0.957, RMSEA = 0.036, SRMR = 0.046) indicating a good model fit. We considered variables including age, income, and work experience in the model to control the marginal effects on the job satisfaction of employees.

The results in Figure 2 reveal that the working environment had a statistically positive effect on perceived fairness (*β* = 0.236, *p* < 0.05) and psychological safety (*β* = 0.206, *p* < 0.01); working conditions also had a statistically positive effect on perceived fairness (*β* = 0.296, *p* < 0.01) and psychological safety (*β* = 0.357, *p* < 0.01), confirming H1, H2, H3, and H4. Simultaneously, health and safety (*β* = 0.163, *p* < 0.01), education and training (*β* = 0.142, *p* < 0.01), and psychological safety (*β* = 0.157, *p* < 0.05) had a significant positive effect on perceived fairness. We also found significant positive correlations between health and safety (*β* = 0.226, *p* < 0.01), education and training (*β* = 0.149, *p* < 0.01), and psychological safety. These findings support H5–H9. Detailed statistic results can be found in Table 6.

The working environment, working conditions, health and safety, education and training are basic physiological, safety, and social needs. Thus, disasters compromise and amplify these basic needs [48]. For example, Ahmad, Donia and Shahzad [52] assert that if organizations can meet the needs of employees and take care of them to make them think the company is ethical, this will lead to a greater sense of psychological safety. Our study validates this result. However, Zhao, et al. [87] propose that the most important factor affecting the psychological-safety status of employees is the leadership style of the organization, especially inclusive leadership, which can significantly improve the psychological safety of employees. This study expands the work of Zhao, Ahmed and Faraz [87] to show that meeting employees’ basic needs can also improve psychological safety, rather than just the influence of leadership.

Moreover, according to the equity theory, improving the working environment, working conditions, health and safety, education and training, and psychological safety can reduce the inputs of couriers, thereby increasing outcomes, and couriers will perceive fairness. Thus, this study is consistent with previous studies on fairness theory [50,63]. However, Al-zawahreh and Al-Madi [63] argue that education and training are employee input, contrary to our findings. Al-zawahreh and Al-Madi [63] define education and training as the ability of employees to serve the company by bringing the education and training they originally received into the company. This study validates that the education and training provided by companies as an outcome of employee perception can improve their perceived fairness, and expands on the research of Al-zawahreh and Al-Madi [63] about fairness theory.

Among the four influencing factors—whether perceived fairness or psychological safety—working conditions of couriers, including income, working hours, benefits, and labor contracts had the greatest positive impact. This finding suggests that couriers’ perceived fairness and psychological safety during the COVID-19 pandemic were more vulnerable to working conditions.

The results in Figure 2 also reveal a significant positive correlation between perceived fairness, psychological safety, and job satisfaction at the 1% significance level. Moreover, standardized coefficients of 0.259 and 0.545 indicate accepting H10 and H11. This finding illustrates that during the COVID-19 pandemic, perceived fairness and psychological safety positively impacted couriers’ job satisfaction. These conclusions are consistent with the results of previous studies [69,75]. Significantly, even though previous research has verified that perceived fairness and psychological safety at work are important factors affecting employee satisfaction, research on labor-intensive businesses under the COVID-19 pandemic is still inadequate. This study used the samples collected during the COVID-19 pandemic, enriching research on infectious diseases faced by employees in labor-intensive enterprises.

Hence, courier companies faced social-sustainability issues in the context of COVID-19 because the perceived fairness and psychological safety of couriers were compromised, resulting in a decline in couriers’ job satisfaction. Therefore, when courier companies try to improve couriers’ job satisfaction, it is necessary to establish a good psychological sense of security by focusing on improving the perceived fairness of couriers.

Moreover, when we compared the control variables (age, income, and work experience) with couriers’ job satisfaction, we found that income positively affected perceived fairness at the 10% significance level. This finding objectively confirms the importance of working conditions. However, this result is unanticipated as the effect of age and work experience on job satisfaction is not significant. Moreover, existing research suggests that the personal pursuits, motivations, and goals of younger, older, new, and longstanding employees are fundamentally different in the same organizational environment [88]. For example, monetary rewards affect job satisfaction among younger employees, while task contributions are more important for older employees [50]. However, it also shows that the theoretical structure of this study predicts couriers’ job satisfaction more accurately than demographics.

### 4.3. Mediation Test

Mediation analysis was employed to explore the indirect effects between the constructs (Table 7). To do so, we conducted the bootstrapping method using 5000 replications with bias-corrected confidence intervals [89]. Overall, the indirect effects between the variable were found to be significant at 95% confidence intervals. Specifically, we found that PS partially mediated the impacts of WE (b_ind_ = 0.032, Boot SE = 0.021, *p* < 0.05), WC (b_ind_ = 0.056, Boot SE = 0.030, *p* < 0.05), HS (b_ind_ = 0.035, Boot SE = 0.020, *p* < 0.05), and ET (b_ind_ = 0.023, Boot SE = 0.015, *p* < 0.05) on PF. Moreover, the results suggested that the indirect effects of WE (b_ind_ = 0.181, Boot SE = 0.050, *p* < 0.001), WC (b_ind_ = 0.287, Boot SE = 0.060, *p* < 0.001), HS (b_ind_ = 0.175, Boot SE = 0.050, *p* < 0.001), and ET (b_ind_ = 0.175, Boot SE = 0.049, *p* < 0.001) on JS were significantly positive. Finally, PF was a partial mediator of the relationship between PS and JS (b_ind_ = 0.040, Boot SE = 0.021, *p* < 0.05).

## 5. Conclusions

### 5.1. Theoretical Contributions

This study makes several significant theoretical contributions to the literature. First, it enriches the literature on social-sustainability practices and employee-level job satisfaction during the COVID-19 pandemic. The COVID-19 pandemic exposed couriers of courier companies to health risks, and increased consumer demand for e-commerce put couriers under greater pressure to work. Coupled with the discomfort of the working environment of the courier company and the limited career development of the courier due to the low entry threshold, it resulted in serious social-sustainability issues for courier companies. This study explored the potential factors that affected the job satisfaction of couriers during the COVID-19 pandemic through social-sustainability practices in courier companies. These factors help address the social-sustainability issues during COVID-19 or similar disasters for courier companies, help improve job satisfaction among couriers, and fill gaps in sustainability practices and job satisfaction research in the corporate community relating to the COVID-19 pandemic.

Second, this study combined Maslow’s hierarchy-of-needs theory, equity theory, and psychological-safety theory to integrate a new theoretical model. We also designed a dedicated measuring scale in conjunction with existing studies to explain the antecedents that affect the job satisfaction of couriers in the COVID-19 pandemic. In addition, this study found that higher levels of perceived fairness and psychological safety contributed to an uptick in couriers’ job satisfaction, consistent with previous research findings [69,75]. Moreover, this study explored the positive relationship between social-sustainability practices at the employee level (i.e., working environment, working conditions, health and safety, education, and training) and psychological safety and perceived fairness.

Third, this study enriches the psychological-safety theory. In existing research on psychological safety, the antecedents affecting psychological safety include supportive leadership behaviors, team characteristics, supportive organizational practices, etc. [68]. We agree with them, but our research expands the psychological-safety theory by validating that meeting the basic needs of employees (environment, treatment, health, and training) can also have a positive impact on employee psychological safety. Meanwhile, Wang, et al. [90] in their research on the safety of employees in oil companies assert that the improvement in employee psychological safety helps to improve the level of their safe behavior. We do not disagree with them, but our findings have enriched the outcomes of the theory of psychological safety by validating the study of couriers during the COVID-19 pandemic to verify that psychological safety can also have a significant impact on employee satisfaction.

Fourth, this study enriches the literature on the equity theory. Previous research on equity theory argued that employees own the educational experience, an input in the working relationship [63]. However, due to couriers’ overall low level of education (as evidenced by demographics), education and training contribute to courier career development. Therefore, in this study, education and training, as outcomes, significantly impact perceived fairness, complementing the study of equity theory in terms of outcomes. In addition, Harrington and Lee [91] asserted that an important factor influencing employee-perceived fairness is psychological contract fulfillment, not anything else. Psychological-contract fulfillment is a vague concept that Harrington and Lee [91] define as the reward that employees and employers expect from the other party. However, working conditions, work environment, health and safety, education, and training are also rewards that employees expect from employers, and this study validates their impact on perceived fairness and refines research on equity theory.

### 5.2. Managerial Implications

Our findings provide some management implications, addressing the social sustainability of courier companies during the COVID-19 pandemic.

First, given the direct impact of the COVID-19 pandemic on labor-intensive businesses such as courier companies, our findings indicate that the companies must ensure the health and safety of their couriers. For example, to prevent the potential spread of COVID-19, companies can regularly disinfect and clean their workplaces and issue free masks to couriers—wearing masks plays a vital role in effectively controlling the spread of COVID-19 [92]. In addition to providing free masks for couriers, promoting the importance of masks can also help more couriers wear masks. Meanwhile, regular COVID-19 screening for couriers (especially those with symptoms) can also make couriers feel healthy and safe working in the company because it tells couriers that the danger is not around them and that the place where they work is safe.

Second, we found that working conditions, work environments, education, and training significantly impacted couriers’ perceived fairness and psychological safety. Improving couriers’ working conditions and working environments and providing more education and training require companies to invest in higher operating costs. However, the profits from increased orders from courier companies during the COVID-19 pandemic can translate into operating costs to address this issue. In addition, courier companies need to recruit more couriers, effectively improving the overall work pressure faced by couriers by reducing each courier’s workload. It is also necessary to upgrade and refine existing remuneration awards and improve their working environment. According to equity theory, this makes couriers perceive that their outcomes have improved. Meanwhile, providing couriers with epidemic prevention guidance during COVID-19 is also an important part of meeting the needs of couriers.

Third, perceived fairness and psychological safety have a significant impact and mediator role, which is a very revealing finding for management. Therefore, companies must enhance couriers’ perceived fairness and psychological safety to enhance job satisfaction. Thus, according to the equity theory, for couriers to perceive fairness, it is necessary to increase the couriers’ outcomes (e.g., compensation, intrinsic rewards, satisfying supervision, etc.) and reduce the couriers’ inputs (e.g., health, time, etc.) [63]. Furthermore, management should give couriers as much care as possible during a disaster such as the COVID-19 pandemic and meet their needs. The company should convince couriers that the company is ethical, thereby improving couriers’ job satisfaction and solving the social-sustainability issues of courier companies.

### 5.3. Limitations and Recommendations

This study has some limitations that could provide direction for follow-up studies. First, this study constructed theoretical models through Maslow’s hierarchy of needs, equity, and psychological-safety theories and explored the antecedents affecting job satisfaction. However, the precursors to employee satisfaction in the HR space are diverse, especially during disasters such as the COVID-19 pandemic. Therefore, we encourage follow-up research on this issue through other human resource theories (e.g., people–organization fit theory, people–environment fit theory, etc.). Second, this study explored the direct (health and safety) and indirect (work pressure) influence of the pandemic on the psychology of corporate employees. Subsequent studies could use other theories (e.g., health belief models, protection motivation theories, etc.) to refine the impact of the COVID-19 pandemic and other similar disasters.

## Figures and Tables

**Table 1:** Theories Explaining Social Sustainability of Courier Companies.

Theory’s Characteristics	Maslow’s Hierarchy-of-Needs Theory	Equity Theory	Psychological-Safety Theory
Paradigm	Psychology	Psychology	Psychology
Basic assumption	There are five levels of human needs, and basic needs are prioritized from the bottom up.	The sense of fairness comes from the ratio of employee inputs to outcomes.	Employees dare to take risks and innovate, and there is no resistance.
Application to model	This theory can explain how to carry out social-sustainability practices by meeting the needs of employees.	This theory could explain how to increase employee satisfaction by improving employee perceived fairness.	Changing theories can illustrate how basic needs affect psychological safety and alter employee satisfaction.

**Table 2:** Scale Development.

Construct	Measurement Items	Sources
Working environment (WE)	WE1. The company provides good light, ventilation and low noise working environment. WE2. The company provides a hygienic working environment during the COVID-19 pandemic. WE3. The company provides leisure facilities. WE4. I can openly discuss the company’s policies and systems.	Ali and Kaur [39] Faulkner and Badurdeen [76] Trmcico, Demmings, Kniel, Wiedmann and Alcaine [1]
Working conditions (WC)	WC1. My income is reasonable. WC2. My working hours are reasonable. WC3. My benefits are reasonable. WC4. The company signed a reasonable labor contract with me.	Tran, Lau and Ong [58]
Health and safety (HS)	HS1. The workplace is hygienic, clean, and injury-free. HS2. The company arranges certain health checks (e.g., organizational medical examinations; screening for COVID-19). HS3. The company provides masks and ensures employees wear them.	Ali and Kaur [39] Rajak and Vinodh [67] Trmcico, Demmings, Kniel, Wiedmann and Alcaine [1]
Education and training (ET)	ET1. The company will train me to improve my job skills. ET2. My skills and knowledge continue to grow. ET3. I can continue to learn, develop and improve. ET4. The company provides guidance to employees on epidemic prevention and practices social distancing during the COVID-19 pandemic.	Ali and Kaur [39] Rajak and Vinodh [67] Trmcico, Demmings, Kniel, Wiedmann and Alcaine [1]
Perceived fairness (PF)	PF1. I have been treated fairly in this organization. PF2. I believe I get a fair reward for my work. PF3. I get a fair benefit from this organization. PF4. The organization makes decisions in a fair manner.	Kim, Lin and Leung [75]
Psychological safety (PS)	PS1. It is safe to take a risk in my workplace. PS2. Even if I make a mistake, my organization will be a certain tolerance for me. PS3. I feel safe in my workplace during the COVID-19 pandemic.	Ahmad, Donia and Shahzad [52] Lee, Swink and Pandejpong [77]
Job satisfaction (JS)	JS1. I am satisfied with my colleagues. JS2. I am satisfied with the supervisor. JS3. I am satisfied with my income. JS4. I am satisfied with my organization.	Yuen, Loh, Zhou and Wong [72] Sung and Hu [78]

**Table 3:** Respondent Demographics.

Items	Category	Frequency (N = 467)	Percentage (%)
Gender	Male	416	89.1
	Female	51	10.9
Age (years)	<25	259	55.4
	25–35	140	30.0
	36–45	68	14.6
	46–55	0	0.0
	>55	0	0.0
Education	High school or below	256	54.8
	Junior college	178	38.1
	Bachelor or above	33	7.1
Monthly income (CNY) (USD 1 = CNY 6.82 *)	<3000	43	9.2
	3000–6999	242	51.8
	7000–10,000	147	31.5
	>10,000	35	7.5
Work experience (years)	<5	256	54.8
	5–10	178	38.1
	>10	33	7.1

Note. * USD to CNY conversion—last updated 21 August 2022, 03:40 UTC.

**Table 4:** Confirmatory Factor Analysis Results.

Construct	Item	λ	t-Value	AVE	CR
Working environment (WE)	WE1	0.790	-	0.564	0.838
	WE2	0.708	14.964 ***		
	WE3	0.757	16.033 ***		
	WE4	0.748	15.844 ***		
Working conditions (WC)	WC1	0.777	15.553 ***	0.587	0.850
	WC2	0.810	16.157 ***		
	WC3	0.753	15.113 ***		
	WC4	0.722	-		
Health and safety (HS)	HS1	0.735	12.837 ***	0.538	0.777
	HS2	0.751	12.966 ***		
	HS3	0.714	-		
Education and training (ET)	ET1	0.728	14.706 ***	0.574	0.843
	ET2	0.746	15.071 ***		
	ET3	0.811	16.206 ***		
	ET4	0.742	-		
Perceived fairness (PF)	PF1	0.805	-	0.666	0.889
	PF2	0.845	20.291 ***		
	PF3	0.816	19.435 ***		
	PF4	0.798	18.874 ***		
Psychological safety (PS)	PS1	0.783	-	0.555	0.789
	PS2	0.709	14.304 ***		
	PS3	0.741	14.886 ***		
Job satisfaction (JS)	JS1	0.774	-	0.574	0.843
	JS2	0.773	16.562 ***		
	JS3	0.754	16.137 ***		
	JS4	0.729	15.553 ***		

Note: *** *p* < 0.001; model fit indices: *χ^2^*= 400.698, *df* = 278, *χ^2^/df* = 1.441 (*p* < 0.05, *df* = 278); CFI = 0.979; GFI = 0.940; TLI = 0.976; RMSEA = 0.031; SRMR = 0.033.

**Table 5:** AVE, Correlations, and Squared Correlations of the Constructs.

	WE	WC	HS	ET	PF	PS	JS
WE	0.564 ^a^	0.264^c^	0.135	0.173	0.352	0.267	0.194
WC	0.514 ^b^	0.587	0.254	0.229	0.445	0.375	0.428
HS	0.367	0.504	0.538	0.088	0.276	0.277	0.135
ET	0.416	0.479	0.296	0.574	0.248	0.181	0.366
PF	0.593	0.667	0.525	0.498	0.666	0.382	0.346
PS	0.517	0.612	0.526	0.426	0.618	0.555	0.452
JS	0.440	0.654	0.368	0.605	0.588	0.672	0.574

Note: ^a^ AVE values are along the main diagonal; ^b^ below main diagonal lists the correlations between constructs; ^c^ squared correlations between the constructs are above the main diagonal.

**Table 6:** Hypothesis test results.

Hypothesis	Path	Path Coefficient	Standard Path Coefficient	*p*-Value	Standard Error	t-Value	Test Result
H1	Working environment → Perceived fairness	0.185	0.236 **	0.000	0.040	4.567	Supported
H2	Working environment → Psychological safety	0.157	0.206 **	0.000	0.042	3.687	Supported
H3	Working conditions → Perceived fairness	0.311	0.296 **	0.000	0.066	4.678	Supported
H4	Working conditions → Psychological safety	0.367	0.357 **	0.000	0.068	5.402	Supported
H5	Health and safety → Perceived fairness	0.167	0.163 **	0.002	0.054	3.094	Supported
H6	Health and safety → Psychological safety	0.226	0.226 **	0.000	0.057	5.402	Supported
H7	Education and training → Perceived fairness	0.159	0.142 **	0.003	0.054	2.957	Supported
H8	Education and training → Psychological safety	0.164	0.149 **	0.004	0.058	2.841	Supported
H9	Psychological safety → Perceived fairness	0.160	0.157 *	0.016	0.067	2.400	Supported
H10	Psychological safety → Job satisfaction	0.593	0.545 **	0.000	0.074	7.997	Supported
H11	Perceived fairness → Job satisfaction	0.272	0.259 **	0.000	0.064	4.272	Supported
							
Controls	Age → Job satisfaction	−0.066	−0.062 ^ns^	0.108	0.041	−1.607	–
	Income → Job satisfaction	0.084	0.063 ^†^	0.099	0.051	1.651	–
	Work experience → Job satisfaction	0.028	0.018 ^ns^	0.639	0.059	0.468	–

Note: ^†^ *p* < 0.1, * *p* < 0.05, ** *p* < 0.01, ^ns^ not significant.

**Table 7:** Bootstrapping Test Results.

	Indirect Effect	Boot SE ^a^	Significance	BLLCI ^b^	BULCI ^c^
WE to PF	0.032	0.021	*	0.003	0.089
WE to JS	0.181	0.050	***	0.093	0.273
WC to PF	0.056	0.030	*	0.010	0.134
WC to JS	0.287	0.060	***	0.172	0.408
HS to PF	0.035	0.020	*	0.005	0.088
HS to JS	0.175	0.050	***	0.075	0.270
ET to PF	0.023	0.015	*	0.003	0.066
ET to JS	0.175	0.049	***	0.028	0.220
PS to JS	0.040	0.021	*	0.008	0.093

Note: ^a^ Boot SE: Bootstrap standard error, ^b^ BLLCI: Bootstrap lower limit confidence interval, ^c^ BULCI: Bootstrap upper limit confidence interval, * *p* < 0.05, *** *p* < 0.001.

## Data Availability

The datasets analyzed during the current study are available from the corresponding author on reasonable request.
